# Data on health risk assessment of fluoride in water distribution network of Iranshahr, Iran

**DOI:** 10.1016/j.dib.2018.08.184

**Published:** 2018-09-12

**Authors:** Majid Radfard, Massuomeh Rahmatinia, Hamed Akbari, Bayram Hashemzadeh, Hesam Akbari, Amir Adibzadeh

**Affiliations:** a Health Research Center, Life style institute, Baqiyatallah University of Medical Sciences, Tehran, Iran; b Department of Environmental Health Engineering, School of Public Health, Iran University of Medical Sciences, Tehran, Iran; c Khoy University of Medical Sciences, Khoy, Iran; d Research Center for Health Sciences, Institute of Health, Department of Environmental Health, School of Health, Shiraz University of Medical Sciences, Shiraz, Iran

**Keywords:** Drinking water, Fluoride, Risk assessment, Iranshahr, Iran

## Abstract

The main of this data was determine the concentrations and health risks of fluoride in 66 drinking water samples collected from villages of the Iranshahr city, Sistan and Baluchestan Province in Iran. Fluoride concentration was measured by the standard SPADNS method. Data indicated that fluoride concentration in drinking water ranged from 0.25 to 1.72 mg L^−1^ and average of fluoride concentration was 0.27 mg L^−1^. The mean estimated daily intake (EDI) values for fluoride in different groups of infants, children, teenagers and adults were 0.0021, 0.0151, 0.0107 and 0.0086 mg/kg, respectively. Also, risk assessment data indicated that hazard quotient (HQ) value of groundwater samples is more than 1 in 6% of groundwater samples in age groups of children and teenagers.

**Specifications table**

**Table:** 

Subject area	Water quality
More specific subject area	Water fluoride
Type of data	Table and Figure
How data was acquired	Spectrophotometer (DR/5000, USA).
Data format	Raw, Analyzed
Experimental factors	Water samples were taken from 66 stations and were stored in polyethylene bottles in a dark place at room temperature until analysis.
Experimental features	The levels of fluoride concentration were determined.
Data source location	Iranshahr region, of Sistan and Baluchestan Province, Iran
Data accessibility	The data are included in this article
Related research article	Yousefi M, Ghoochani M, Mahvi AH. Health risk assessment to fluoride in drinking water of rural residents living in the Poldasht city, Northwest of Iran. Ecotoxicology and environmental safety. 2018; 148:426-30.

**Value of the data**

• Knowledge of fluoride level in potable groundwater is important for health care personnel and policymaker.
• Based on data, the fluoride concentration in 98% of water samples were less than the maximum permissible limits (1.5 mg/L) according WHO guidelines.
• Health risk assessment and data analysis indicated that HQ value was less than 1 in 94% of samples in all age groups and HQ value was more than 1 in 6% of samples in age groups of children and teenagers, so should be selected a suitable resource of drinking water for this age group.
• Base on the data, DE fluoridation of drinking water could be recommended in fluorotic rural area with high fluoride concentrations.

## 1 Data

**Fig. 1:**
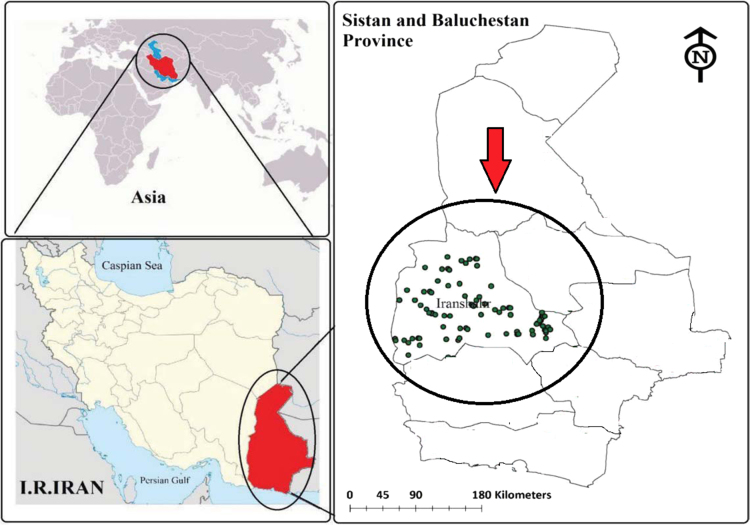
Location of water sampling in Iranshahr city, Sistan and Baluchistan province, Iran.

Fig. 1 shows location of the water sampling in Iranshahr city, Sistan and Baluchistan province, Iran.

**Fig. 2:**
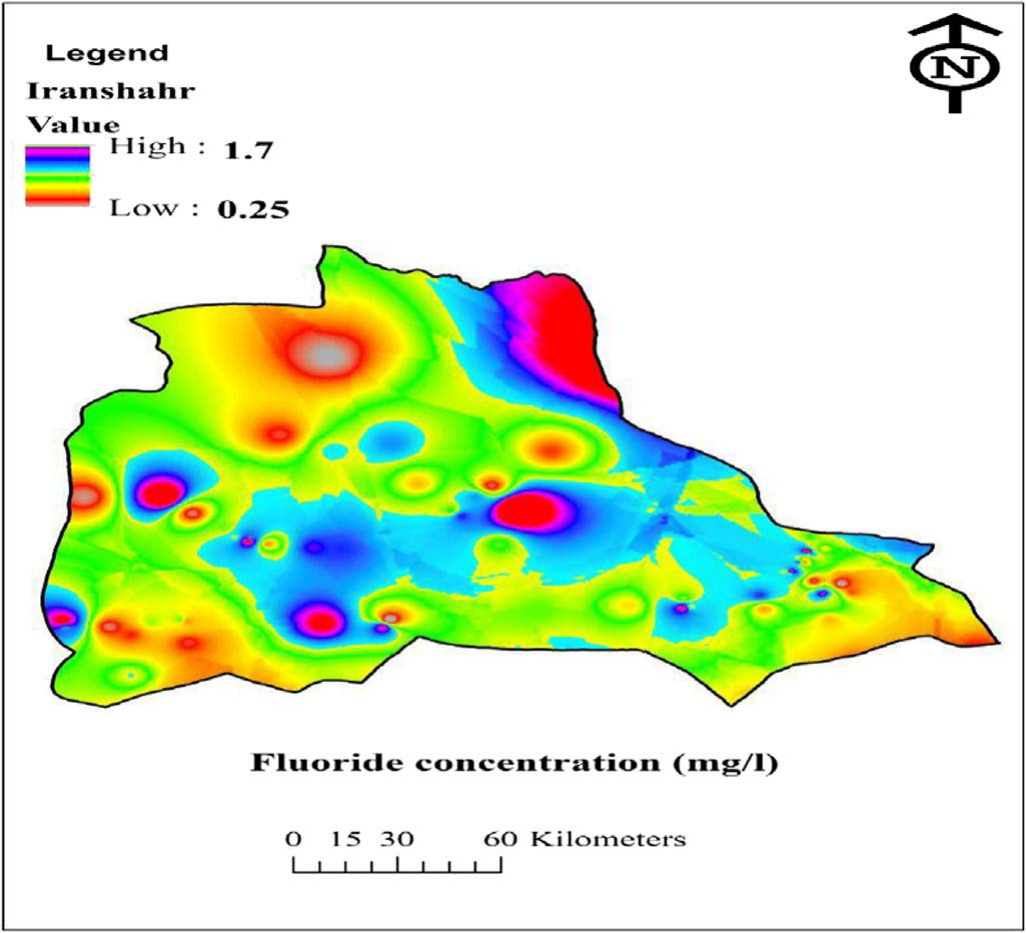
Dispersion of F concentration (mg L^−1^) by GIS software.

And Fig. 2 Dispersion of fluoride by GIS software. the parameters used to in this data for health exposure assessment in drinking water. Fluoride concentration, EDI and HQ for the four populations of water consumers in the data have been shown in Tables 1 and 2, respectively.

**Table 1:** Parameters used to in this data for health exposure assessment in drinking water [1], [2], [3], [4], [5], [6], [7].

Parameter	Risk exposure factors	Values for groups				Unit
		Infants	Children	Teenagers	Adults	
Fluoride	C_f_	–	–	–	–	mg/L
	C_d_	0.08	0.85	2	2.5	Liter/day
	B_w_	10	15	50	78	Kg
	RfD	0.06	0.06	0.06	0.06	mg/kg.day

**Table 2:** The fluoride concentration in drinking water and EDI and HQ for the four populations of water consumers.

Nos	Fluoride	Infants	EDI	Teenagers	Adults	Infants	HQ	Teenagers	Adults
	concentration		Children				Children		
1	0.380	0.0030	0.0215	0.0152	0.0122	0.0507	0.3589	0.2533	0.2030
2	0.590	0.0047	0.0334	0.0236	0.0189	0.0787	0.5572	0.3933	0.3152
3	0.770	0.0062	0.0436	0.0308	0.0247	0.1027	0.7272	0.5133	0.4113
4	0.700	0.0056	0.0397	0.0280	0.0224	0.0933	0.6611	0.4667	0.3739
5	0.420	0.0034	0.0238	0.0168	0.0135	0.0560	0.3967	0.2800	0.2244
6	0.510	0.0041	0.0289	0.0204	0.0163	0.0680	0.4817	0.3400	0.2724
7	0.810	0.0065	0.0459	0.0324	0.0260	0.1080	0.7650	0.5400	0.4327
8	0.500	0.0040	0.0283	0.0200	0.0160	0.0667	0.4722	0.3333	0.2671
9	0.780	0.0062	0.0442	0.0312	0.0250	0.1040	0.7367	0.5200	0.4167
10	0.630	0.0050	0.0357	0.0252	0.0202	0.0840	0.5950	0.4200	0.3365
11	0.610	0.0049	0.0346	0.0244	0.0196	0.0813	0.5761	0.4067	0.3259
12	0.350	0.0028	0.0198	0.0140	0.0112	0.0467	0.3306	0.2333	0.1870
13	0.530	0.0042	0.0300	0.0212	0.0170	0.0707	0.5006	0.3533	0.2831
14	0.410	0.0033	0.0232	0.0164	0.0131	0.0547	0.3872	0.2733	0.2190
15	0.290	0.0023	0.0164	0.0116	0.0093	0.0387	0.2739	0.1933	0.1549
16	0.540	0.0043	0.0306	0.0216	0.0173	0.0720	0.5100	0.3600	0.2885
17	0.940	0.0075	0.0533	0.0376	0.0301	0.1253	0.8878	0.6267	0.5021
18	0.590	0.0047	0.0334	0.0236	0.0189	0.0787	0.5572	0.3933	0.3152
19	0.690	0.0055	0.0391	0.0276	0.0221	0.0920	0.6517	0.4600	0.3686
20	0.900	0.0072	0.0510	0.0360	0.0288	0.1200	0.8500	0.6000	0.4808
21	0.510	0.0041	0.0289	0.0204	0.0163	0.0680	0.4817	0.3400	0.2724
22	1.720	0.0138	0.0975	0.0688	0.0551	0.2293	1.6244	1.1467	0.9188
23	1.070	0.0086	0.0606	0.0428	0.0343	0.1427	1.0106	0.7133	0.5716
24	1.050	0.0084	0.0595	0.0420	0.0337	0.1400	0.9917	0.7000	0.5609
25	0.970	0.0078	0.0550	0.0388	0.0311	0.1293	0.9161	0.6467	0.5182
26	0.950	0.0076	0.0538	0.0380	0.0304	0.1267	0.8972	0.6333	0.5075
27	0.600	0.0048	0.0340	0.0240	0.0192	0.0800	0.5667	0.4000	0.3205
28	0.690	0.0055	0.0391	0.0276	0.0221	0.0920	0.6517	0.4600	0.3686
29	0.420	0.0034	0.0238	0.0168	0.0135	0.0560	0.3967	0.2800	0.2244
30	0.530	0.0042	0.0300	0.0212	0.0170	0.0707	0.5006	0.3533	0.2831
31	0.440	0.0035	0.0249	0.0176	0.0141	0.0587	0.4156	0.2933	0.2350
32	0.310	0.0025	0.0176	0.0124	0.0099	0.0413	0.2928	0.2067	0.1656
33	0.870	0.0070	0.0493	0.0348	0.0279	0.1160	0.8217	0.5800	0.4647
34	1.220	0.0098	0.0691	0.0488	0.0391	0.1627	1.1522	0.8133	0.6517
35	0.75	0.0060	0.0425	0.0300	0.0240	0.1000	0.7083	0.5000	0.4006
36	0.76	0.0061	0.0431	0.0304	0.0244	0.1013	0.7178	0.5067	0.4060
37	0.62	0.0050	0.0351	0.0248	0.0199	0.0827	0.5856	0.4133	0.3312
38	0.28	0.0022	0.0159	0.0112	0.0090	0.0373	0.2644	0.1867	0.1496
39	1.03	0.0082	0.0584	0.0412	0.0330	0.1373	0.9728	0.6867	0.5502
40	0.53	0.0042	0.0300	0.0212	0.0170	0.0707	0.5006	0.3533	0.2831
41	1.25	0.0100	0.0708	0.0500	0.0401	0.1667	1.1806	0.8333	0.6677
42	0.52	0.0042	0.0295	0.0208	0.0167	0.0693	0.4911	0.3467	0.2778
43	0.62	0.0050	0.0351	0.0248	0.0199	0.0827	0.5856	0.4133	0.3312
44	0.57	0.0046	0.0323	0.0228	0.0183	0.0760	0.5383	0.3800	0.3045
45	0.73	0.0058	0.0414	0.0292	0.0234	0.0973	0.6894	0.4867	0.3900
46	0.37	0.0030	0.0210	0.0148	0.0119	0.0493	0.3494	0.2467	0.1976
47	0.32	0.0026	0.0181	0.0128	0.0103	0.0427	0.3022	0.2133	0.1709
48	0.54	0.0043	0.0306	0.0216	0.0173	0.0720	0.5100	0.3600	0.2885
49	0.54	0.0043	0.0306	0.0216	0.0173	0.0720	0.5100	0.3600	0.2885
50	0.39	0.0031	0.0221	0.0156	0.0125	0.0520	0.3683	0.2600	0.2083
51	0.76	0.0061	0.0431	0.0304	0.0244	0.1013	0.7178	0.5067	0.4060
52	0.43	0.0034	0.0244	0.0172	0.0138	0.0573	0.4061	0.2867	0.2297
53	0.54	0.0043	0.0306	0.0216	0.0173	0.0720	0.5100	0.3600	0.2885
54	0.83	0.0066	0.0470	0.0332	0.0266	0.1107	0.7839	0.5533	0.4434
55	0.36	0.0029	0.0204	0.0144	0.0115	0.0480	0.3400	0.2400	0.1923
56	0.58	0.0046	0.0329	0.0232	0.0186	0.0773	0.5478	0.3867	0.3098
57	0.37	0.0030	0.0210	0.0148	0.0119	0.0493	0.3494	0.2467	0.1976
58	0.42	0.0034	0.0238	0.0168	0.0135	0.0560	0.3967	0.2800	0.2244
59	0.25	0.0020	0.0142	0.0100	0.0080	0.0333	0.2361	0.1667	0.1335
60	0.5	0.0040	0.0283	0.0200	0.0160	0.0667	0.4722	0.3333	0.2671
61	0.84	0.0067	0.0476	0.0336	0.0269	0.1120	0.7933	0.5600	0.4487
62	0.69	0.0055	0.0391	0.0276	0.0221	0.0920	0.6517	0.4600	0.3686
63	0.93	0.0074	0.0527	0.0372	0.0298	0.1240	0.8783	0.6200	0.4968
64	0.54	0.0043	0.0306	0.0216	0.0173	0.0720	0.5100	0.3600	0.2885
65	0.51	0.0041	0.0289	0.0204	0.0163	0.0680	0.4817	0.3400	0.2724
66	0.75	0.0060	0.0425	0.0300	0.0240	0.1000	0.7083	0.5000	0.4006
Mean	0.27	0.0021	0.0151	0.0107	0.0086	0.0356	0.2522	0.1781	0.1427
Min	0.25	0.0020	0.0142	0.0100	0.0080	0.0333	0.2361	0.1667	0.1335
Max	1.72	0.0138	0.0975	0.0688	0.0551	0.2293	1.6244	1.1467	0.9188
SD	0.27	0.0021	0.0151	0.0107	0.0086	0.0356	0.2522	0.1781	0.1427

## 2 Experimental design, materials and methods

### 2.1 Description of study area

Iranshahr city is located in Sistan and Baluchistan province of Iran between the latitudes 27°12′ N and Longitudes 60° 41′ E. According to the demographic information of Iran, this city encompassed with an area 30,200 km^2^. Iranshahr city has a warm and dry climate with an annual mean temperature of 26.5 °C [8], [9], [10].

### 2.2 Determination of fluoride concentration in drinking water

For this data, 66 samples were taken from drinking water resources from villages during 12 months (2016). For this purpose, polyethylene bottles washed twice with deionized water and used for sampling. The water samples were transported to the laboratory in 4° and stored in a dark place at room temperature until analysis. Fluoride concentration of water samples were analyzed by SPADNS method using UV-visible Spectrophotometer (DR/5000, USA) [11], [12], [13], [14], [15], [16], [17], [18], [19], [20], [21].

### 2.3 Risk assessment of fluoride

In order to estimate the probability of adverse health effects it is necessary to assess human health risk. So, the quantitative health risk assessment of fluoride through consumption of drinking water was evaluated in rural population of Iranshahr city. For this purpose, water samples were taken from village areas. We divided population into four age groups based on physiological and behavioral differences according to study by Yousefi et al. (2017) as fallow: infants (less than 2 years), children (2 to <6 years), teenagers (6 to <16 years) and adults (≥ 16 years). The daily exposure to fluoride was calculated in these groups using Eq. (1) [2]:

(1) $$EDI=\frac{C_{f}\times C_{d}}{B_{w}}$$

EDI: Estimation of daily fluoride consumption
C_f_: Fluoride concentration in drinking water (mg/L)
C_d_: Average daily drinking water intake
B_w_: body weight (Kg)

Water consumption and body weight data were measured based on a questionnaire that was asked from target groups (infants, children, adolescents and adults). The average water consumption rates in infants (0–2 years old), children (2–6 years old), teenagers (6–16 years old) and adults (≥ 16 years old) were 0.08, 0.85, 2 and 2.5 L day^−1^, respectively. Body weights of target groups were considered 10, 15, 50 and 78 kg, respectively. HQ is the non-carcinogenic risk of fluoride to human health that was calculated using Eq. (2).

(2) $$HQ=\frac{EDI}{RFD}$$

EDI: Estimated Daily intake (mg/kg d)
RFD: reference dose of fluoride (mg/Kg/day)

The reference dose for fluoride is (0.06 mg kg^−1^ d^−1^).

A value of HQ less than one indicates a negligible risk of non-carcinogenic effects and HQ higher than one indicates a significant risk level [22], [23], [24], [25].

## References

[1] Mirzabeygi M., Yousefi M., Soleimani H., Mohammadi A.A., Mahvi A.H., Abbasnia A. (2018). The concentration data of fluoride and health risk assessment in drinking water in the Ardakan city of Yazd province, Iran. Data Brief.

[2] Yousefi M., Ghoochani M., Mahvi A.H. (2018). Health risk assessment to fluoride in drinking water of rural residents living in the Poldasht city, Northwest of Iran. Ecotoxicol. Environ. Saf..

[3] Asghari F.B., Mohammadi A.A., Aboosaedi Z., Yaseri M., Yousefi M. (2017). Data on fluoride concentration levels in cold and warm season in rural area of Shout (West Azerbaijan, Iran). Data Brief.

[4] Radfard M., Yunesian M., Nabizadeh R., Biglari H., Nazmara S., Hadi M. (2018). Drinking water quality and arsenic health risk assessment in Sistan and Baluchestan, Southeastern Province, Iran. Hum. Ecol. Risk Assess.: Int. J..

[5] Abbasnia A., Ghoochani M., Yousefi N., Nazmara S., Radfard M., Soleimani H. (2018). Prediction of human exposure and health risk assessment to trihalomethanes in indoor swimming pools and risk reduction strategy. Hum. Ecol. Risk Assess.: Int. J..

[6] Mirzabeygi M., Abbasnia A., Yunesian M., Nodehi R.N., Yousefi N., Hadi M. (2017). Heavy metal contamination and health risk assessment in drinking water of Sistan and Baluchistan, Southeastern Iran. Hum. Ecol. Risk Assess.: Int. J..

[7] Abbasnia A., Alimohammadi M., Mahvi A.H., Nabizadeh R., Yousefi M., Mohammadi A.A. (2018). Assessment of groundwater quality and evaluation of scaling and corrosiveness potential of drinking water samples in villages of Chabahr city, Sistan and Baluchistan province in Iran. Data Brief.

[8] Abbasnia A., Yousefi N., Mahvi A.H., Nabizadeh R., Radfard M., Yousefi M. (2018). Evaluation of groundwater quality using water quality index and its suitability for assessing water for drinking and irrigation purposes: case study of Sistan and Baluchistan province (Iran). Hum. Ecol. Risk Assess.: Int. J..

[9] Abbasnia A., Radfard M., Mahvi A.H., Nabizadeh R., Yousefi M., Soleimani H. (2018). Groundwater quality assessment for irrigation purposes based on irrigation water quality index and its zoning with GIS in the villages of Chabahar, Sistan and Baluchistan, Iran. Data Brief.

[10] Mohammadi A.A., Yousefi M., Yaseri M., Jalilzadeh M., Mahvi A.H. (2017). Skeletal fluorosis in relation to drinking water in rural areas of West Azerbaijan, Iran. Sci. Rep..

[11] Yousefi M., Yaseri M., Nabizadeh R., Hooshmand E., Jalilzadeh M., Mahvi A.H. (2018). Association of hypertension, body mass index, and waist circumference with fluoride intake; water drinking in residents of fluoride endemic areas, Iran. Biol. Trace Elem. Res..

[12] Yousefi M., Arami S.M., Takallo H., Hosseini M., Radfard M., Soleimani H. (2018). Modification of pumice with HCl and NaOH enhancing its fluoride adsorption capacity: kinetic and isotherm studies. Hum. Ecol. Risk Assess.: Int. J..

[13] Takdastan A., Mirzabeygi M., Yousefi M., Abbasnia A., Khodadadia R., Soleimani H. (2018). Neuro-fuzzy inference system Prediction of stability indices and Sodium absorption ratio in Lordegan rural drinking water resources in west Iran. Data Brief.

[14] Neisi A., Mirzabeygi M., Zeyduni G., Hamzezadeh A., Jalili D., Abbasnia A. (2018). Data on fluoride concentration levels in cold and warm season in City area of Sistan and Baluchistan Province, Iran. Data Brief.

[15] Djahed B., Taghavi M., Farzadkia M., Norzaee S., Miri M. (2018). Stochastic exposure and health risk assessment of rice contamination to the heavy metals in the market of Iranshahr, Iran. Food Chem. Toxicol..

[16] Miri M., Bhatnagar A., Mahdavi Y., Basiri L., Nakhaei A., Khosravi R. (2018). Probabilistic risk assessment of exposure to fluoride in most consumed brands of tea in the Middle East. Food Chem. Toxicol..

[17] Fallahzadeh R.A., Khosravi R., Dehdashti B., Ghahramani E., Omidi F., Adli A. (2018). Spatial distribution variation and probabilistic risk assessment of exposure to chromium in ground water supplies; a case study in the east of Iran. Food Chem. Toxicol..

[18] Fallahzadeh R.A., Miri M., Taghavi M., Gholizadeh A., Anbarani R., Hosseini-Bandegharaei A. (2018). Spatial variation and probabilistic risk assessment of exposure to fluoride in drinking water. Food Chem. Toxicol..

[19] Akbari H. (2018). Data on investigating the nitrate concentration levels and quality of bottled water in Torbat-e Heydarieh, Khorasan Razavi province, Iran. Data Brief.

[20] Babaeia A. (2018). Data on groundwater quality, scaling potential and corrosiveness of water samples in Torbat-e-Heydariyeh rural drinking water resources, Khorasan-e-Razavi province, Iran. Data Brief.

[21] Heidarinejad Z. (2018). Data on quality indices of groundwater resource for agricultural use in the Jolfa, East Azerbaijan, Iran. Data Brief.

[22] Jafari K. (2018). Data on microbiological quality assessment of rural drinking water supplies in Tiran County, Isfahan province, Iran. Data Brief.

[23] Jalili D. (2018). Data on nitrate-nitrite pollution in the groundwater resources a Sonqor plain in Iran. Data Brief.

[24] Soleimani H. (2018). Data on drinking water quality using water quality index (WQI) and assessment of groundwater quality for irrigation purposes in Qorveh&Dehgolan, Kurdistan, Iran. Data Brief.

[25] Abbasnia A. (2018). Prediction of human exposure and health risk assessment to trihalomethanes in indoor swimming pools and risk reduction strategy. Hum. Ecol. Risk Assess.: Int. J..

